# Evaluation of nutritional status in children with refractory epilepsy

**DOI:** 10.1186/1475-2891-5-14

**Published:** 2006-04-26

**Authors:** S Bertoli, S Cardinali, P Veggiotti, C Trentani, G Testolin, A Tagliabue

**Affiliations:** 1 International Centre for the Assessment of Nutritional Status (ICANS), University of Milan, Italy; 2 Human Nutrition and Eating Disorders Research Centre, University of Pavia, Italy; 3 Child Neuropsychiatry Department, Casimiro Mondino Foundation, University of Pavia, Italy

## Abstract

**Background:**

children affected by refractory epilepsy could be at risk of malnutrition because of feeding difficulties (anorexia, chewing, swallowing difficulties or vomiting) and chronic use of anticonvulsants, which may affect food intake and energy metabolism. Moreover, their energy requirement may be changed as their disabilities would impede normal daily activities. The aim of the present study was to evaluate nutritional status, energy metabolism and food intake in children with refractory epilepsy.

**Methods:**

17 children with refractory epilepsy (13 boys and 4 girls; mean age 9 ± 3,2 years; Body Mass Index 15,7 ± 3,6) underwent an anthropometric assessment, body composition evaluation by dual-energy X-ray absorptiometry, detailed dietetic survey and measurement of resting energy expenditure by indirect calorimetry. Weight-for-age, height-for-age (stunting) and weight-for-height (wasting) were estimated compared to those of a reference population of the same age.

**Results:**

40% of children were malnourished and 24% were wasted. The nutritional status was worse in the more disabled children. Dietary intake resulted unbalanced (18%, 39%, 43% of total daily energy intake derived respectively from protein, lipid and carbohydrate). Adequacy index [nutrient daily intake/recommended allowance (RDA) × 100] was < 60% for calcium iron and zinc.

**Conclusion:**

many children with refractory epilepsy would benefit from individual nutritional assessment and management as part of their overall care.

## Background

Refractory epilepsy (RE) is a condition in which seizures do not respond to first and second-line anticonvulsant drug therapy. Despite the use of new antiepileptic drugs (many antiepileptic drugs have been marketed in the last decade), refractory epilepsy occurs in approximately 20–30% of patients with epilepsy probably due to the multiple pathogenetic mechanisms underlying refractoriness [1].

Drug-resistant epilepsy is frequent in several disorders such as hereditary metabolic or degenerative disorders, cerebral palsy, severe myoclonic epilepsy of infancy, brain injuries/malformations, Lennox-Gastaut syndrome [2]. Though epilepsy itself does not cause neurological deterioration, the evolution of refractory epilepsy does, since patients are submitted to multiple drug treatments which lead to neurological deterioration in children affected by RE. This is characterised by cognitive decline, motorial problems and behaviour disorders (attention reduction, problems of social relationships and problems of conduct) and leads to disabled children [3,4].

Several authors showed that feeding difficulties and malnutrition are common in disabled children: intake may be reduced because of anorexia, chewing and swallowing difficulties, or vomiting [5]. Moreover, most of the commonly used anticonvulsants influence nutritional status. In particular, some drugs affect the regulation of energy balance and appetite with consequent loss (topiramate) or gain (carbamazepina, valproate) of body weight [6-8]. Phenytoin, phenobarbitone, and carbamazepine can interfere with vitamin D metabolism and increase the risk of osteopenia and osteoporosis [9]. Dahl et al [10] found an altered nutritional status in 52 % of children with moderate or severe cerebral palsy: 43% were underweight and 9% were overweight compared with reference values of healthy children. Undernourished disabled children had significantly lower height for age, weight for height, triceps skinfold thickness and upper-arm circumference than healthy children. A Cross-sectional analyses in a large cohort of disabled children showed that their energy and nutrient intake were lower in comparison with recommended values [11].

Since children with RE gradually become disabled it could be assumed that this state is associated with malnutrition being linked to feeding difficulties, to the wrong choice of foods and to changes in energy requirement due to physical inactivity and drugs. This inadequate nutritional status would then worsen the children's health, in particular their immunity status.

The aims of our study were to evaluate nutritional status, energy metabolism and food intake in children affected by refractory epilepsy.

## Methods

### Subjects

All the children with RE treated at the Child Neuropsychiatry Department, Casimiro Mondino Foundation (Pavia, Italy) (from 1995 to 2000) were invited to participate, except for those affected by diseases causing significant nutritional status impairment (neoplasia, chronic infections), or changes in energy metabolism (hyper-hypothyroidism), or treated with special diets (diabetes, phenylketonuria). Patients on enteral tube or parenteral feeding were also excluded. All children had to be free from acute infections and were being treated only with antiepilectic drugs at the time of the study.

Seventeen children with RE, (13 boys and 4 girls) mean age 9.06 ± 3.17, range 3–16 years were enrolled and studied. The Research ethics board approval was obtained and a consent form was signed by all parents/caregivers before the beginning of the study.

### Experimental procedure

The assessment of nutritional status and energy metabolism was conducted at the International Centre for the assessment of Nutritional Status (ICANS), University of Milan. On the same morning anthropometric measurements and resting energy expenditure were evaluated. Dual-energy X-ray absorptiometry (DXA) was performed on a sub sample of children. The evaluation of food intake was completed at the Human Nutrition and Eating Disorders Research Centre, University of Pavia in all children.

### Anthropometric measurements

Anthropometric measurements were taken by the same operator, according to conventional criteria and measuring procedures [12]. Body Weight (BW, Kg) and Body Height (BH, cm) were measured to the nearest 100 g and 0.5 cm respectively. When the child was not able to maintain the erect position the BW was assessed measuring the parent's weight with the child in their arms and subtracting the weight of parent and the BH was measured using supine length.

Body Mass Index (BMI) was calculated using the formula: BMI (Kg/m^2^) = BW (Kg)/BH^2 ^(m^2^). Triceps and subscapular skinfold thickness measurements, were used to provide an estimate of total body fat and were measured as proposed by Lohman et al [12] by means of to Holtain LTD caliper. All the measurements were performed on the non-dominant side of the body. They were made in triplicate for all sites and the average of the three values was calculated for subsequent analysis.

The BW and BH were compared with the standards for linear growth derived from the Tanner-Whitehouse growth charts [13] and the percentage of ideal BW (%BW) and ideal BH (%BH) for sex and age at 50^th ^percentile was calculated. Cut-off point of malnutritional status was identified at 80% of the weight for age value. According to Waterlow classification [14] children were defined as "stunted " when their %BH was lower than 80% compared with the reference height for age. BW was also espressed as a percentage of ideal weight for height, sex, and age (%IBW-H) [15]. This index is used regularly as a measure of nutritional status in children affected by several diseases such as cystic fibrosis [16], liver disease [17] and tumors [18]. Ideal body weights for height was derived by comparing actual weight with the 50^th ^centile weight for a child of the same height who is on the 50^th ^centile for height [14]. Children were classified as "wasted" when their %IBW-H was lower than 90% compared with reference values [14].

Skinfold thickness measurements, were compared with the reference standards proposed by Rolland-Cachera [19] and were expressed as a percentage of ideal value for sex and age at 50^th ^percentile.

### Body composition assessment

Measurements of fat mass (FM, Kg), fat free mass (FFM, Kg) and bone mineral content (BMC, g) was performed with a Lunar DPX-IQ scanner (Lunar Corp, Madison, WI) equipped with a paediatric software (version 4.6 b). Total body scans were performed with subjects in the supine position. The entire body of each subject was scanned, beginning at the top of the head, with the "medium t" scan mode. The mean measurement time was 15 min; radiation exposure was < 7 μSv. Daily quality-assurance tests were performed according to the manufacturer's directions. All scans were performed and analysed by the same operator.

### Resting energy expenditure

Resting energy expenditure (REE) was estimated by indirect calorimeter using an open-circuit ventilated-hood system (Sensor Medics 29, Anaheim, CA). All measurements were made in post-absorptive state (12–14 h fast) in a thermoneutral environment (24–26°C) and with no external stimulation. The calorimeter was calibrated at the beginning of each test with two reference gas mixtures (26% O_2 _and 74 % N_2 _the first, 16% O_2_, 4.09% CO_2 _and 79.91% N_2 _the second). The children were rested for a least 20 min before the measurements which were on subjects awake and supine. Approximately 30 min of respiratory gas exchange data were collected. The first 5–10 min of data were discarded, as recommended by Isbell et al [20]. This allowed the children time to acclimatise to the canopy and instrument noise. The average of the last 20 min of measurements was used to determine 24 h REE according to standard abbreviated Weir equation [21]:

REE (Kcal/day) = [3.941 VO_2 _(mL/min) +1.106 VCO_2 _(mL/min)] * 1.44.

### Dietary intake data

A seven days food diary was used to collect dietary food intake. Parents were trained by dietician in food recording procedures; when the food record was completed, the family came to the Human Nutrition and Eating Disorders Research Centre, and each day of the food record was assessed for completeness by a trained dietician. Incomplete days were excluded from the nutrient analysis. Food records were coded and analysed using the Dieta Ragionata software (Esi Stampa Medica srl, Milano, Italiy). Total energy intake (EI, kcal), proteins, lipids, carbohydrates, some minerals and vitamins (calcium, phosphate, potassium, iron, zinc, copper, tiamin, riboflavin, niacin, vitamin A vitamin C) were calculated and compared with European Recommended Dietary Allowances for sex and age [22]. The adequacy index was calculated according to the following formula:

daily nutrient intake (g)/recommended allowance (g) for sex and age * 100.

### Statistical analysis

Results are expressed as mean ± SD. *t-*tests were used to compare observed with predicted variables in RE children. Adequacy index was calculated to compare the macro and micro nutrient intake with recommended allowance values [22]. Pearson correlation analysis was used to investigate relations between variables; significance is defined as p < 0.05. Statistical analysis was done using Statistica for Windows version 4.5 (StatSoft).

## Results and discussion

**Figure 1:**
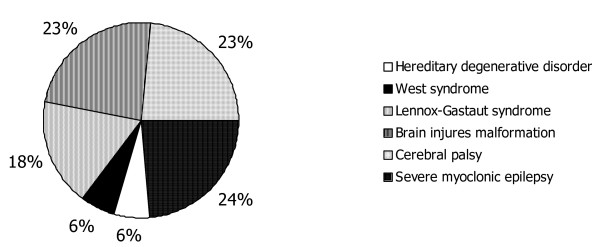
neurological diseases in RE children group (n = 17).

Children's neurological diseases are show in Figure 1.

All children were mildly or severely mentally retarded and had physical developmental delay. Neurological impairment was classified according to difficulty with mobility which was graded as mild (little or no difficulty walking), moderate (difficulty walking but does not need aids or a helper), or severe (needs aids and/or helper or cannot walk). Only 29% of the children had a mild or not difficulty walking whereas 41% had a severe grade. 35% of children presented feeding problems (chewing and swallowing difficulties) and 70% presented an impairment of self-feeding skills.

The anthropometric characteristics of the children are shown in Table 1. Their mean BW and BH were lower but not significantly different from normal values for sex and age. When we consider individual values, we can observe that seven patients (41,2%) had %BW lower than 80% and can therefore be classified as malnourished. Only one patient had %BW higher than 120%. No patient was stunted. IBW-H for sex and age was found lower but not significantly different in comparison with reference value. Four patients (24,0%) had %IBW-H less than 90 % (wasted) and one patient had a % IBW-H higher than 120%.

**Table 1:** nutritional status and resting energy expenditure in RE children (n = 17).

	Mean (SD)	Range
BMI (kg/m2)	15,7 (3,5)	10,4–24,9
%BW	90,0 (29,0)	45,5–175,7
%BH	96,0 (6,6)	80,4–105,7
%IBW-H	96,0 (19,7)	65.0–147,4
% REE	94,8 (26,3)	51,0–132,0
REE/BW (kcal/kg)	38.5 (8.6)	27.2–49.0
Respiratory Quotient	0.83 (0.09)	0.75–0.91

BMI: body mass index

%BW: percentage of ideal body weight for sex and age

%BH: percentage of ideal body height for sex and age

%IBW-H: percentage of ideal weight for height, sex and age

%REE: percentage of measured REE/predicted REE

Mean values of arm circumference (19,52 ± 4,08) and subscapular thickness (6,74 ± 2,76) were found respectively 6% and 4% lower than reference value for sex and age [23] (mean value of arm circumference 20,84 ± 2,67; mean value of subscapular thickness 7,50 ± 2,10).

On the contrary mean values of triceps thickness (9,63 ± 6,46) were 20% higher than reference values for sex and age (8,04 ± 1,99) [24]. Three patients had triceps thickness values lower than third percentile.

The degree of neurological impairment and the presence of feeding problems was correlated with %BW (r^2 ^= 0,26 and r^2 ^= 0,10 respectively). The same results were achieved using, as an index of the nutritional status, the %IBW-H (r^2 ^= 0,32 and r^2 ^= 0,17 respectively). This data suggest that the degree of disability itself influences greatly the nutritional status of the children with RE.

Mean REE was 5% lower than predicted values with a great variation between subjects (range: from - 49% to + 32%).

**Figure 2:**
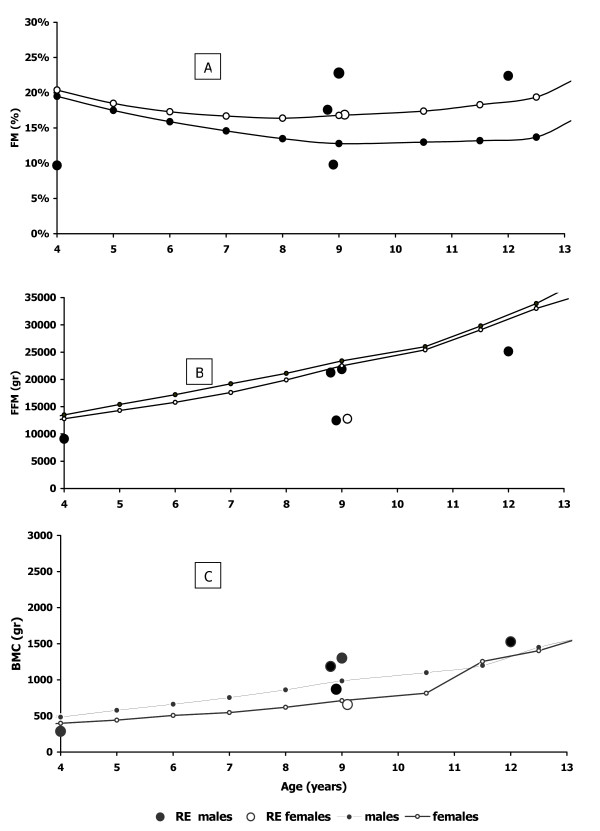
body composition age-related difference in fat mass (A), lean body mass (B) and bone mineral content (C) in RE patients (n = 6) in comparison to male and female reference values.

With regard to body composition, dual-energy X-ray absorptiometry (DXA) was made in a subgroup of children (n = 6). Figure 2 shows age-related differences in fat mass, fat free mass and bone mineral content in RE patients in comparison to male and female reference value [25,26]

All wasted patients undergoing DXA evaluation showed a severe reduction in fat mass and an increased fat mass (in comparison with sex and age reference values) was found in 3 patients with normal body weight indexes (%BW 97,0% ± 2,6 and %IBW-H 102,2 ± 11,1)

Pearson correlations between %FM from DXA and anthropometric indexes were significant (r^2 ^= 0,79 %FM vs %BW; r^2 ^= 0,70 %FM vs %IBW-H).

**Figure 3:**
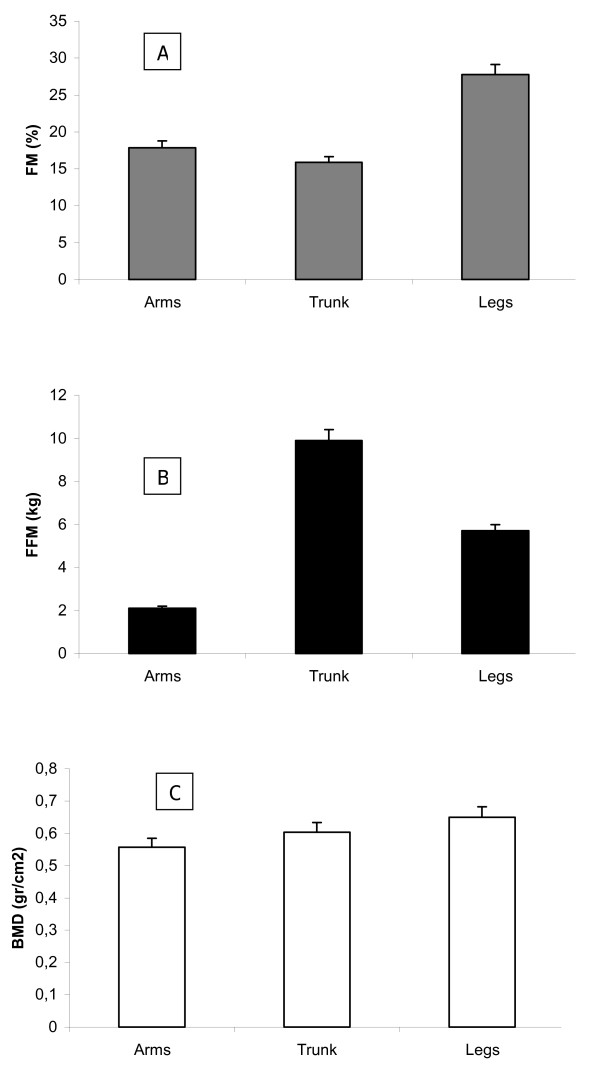
fat mass (A), fat free mass (B) and bone mineral density (C) regional distribution in RE patients (n = 6).

The fat mass distribution appeared modified with a prevailing increase of FM in the limbs (Figure 3A).

FFM was lower than reference values in all children and there was a strong correlation between FFM from DXA and anthropometric indexes (r^2 ^= 0,86 FFM (kg) vs %BW; r^2 ^= 0,95 FFM (kg) vs %IBW-H). Moreover, there appeared a change in FFM distribution with a reduction in the limbs (Figure 3B). Children with more severe neurological impairment had the lowest amount of FFM (r^2 ^= 0,30).

Mean BMD was 0,827 ± 0,128 with mean z-score -0,187 ± 1,36. In particular 3 children were osteopenic and 1 patient presented osteoporosis at the time of the measurements.

Mean daily energy intake (EI) was 1334 ± 295 kcal, different (p = 0.021) from the recommended energy intake for age and sex (1618 ± 358 kcal) [27] Protein intake for body weight was 2.77 ± 1.03 g/kg.

Fat intake average was 39,4 ± 8,3 % and mean intake of saturated acids fatty 11.08 ± 3.78 % of energy intake. Cholesterol intake was 153.40 ± 93.58 mg/die.

The percentage of total energy intake derived from total carbohydrate was 42,7 ± 6,7, greatly lower than recommended values. Complex carbohydrates represented only 19% of total energy intake. Mean daily fibre intake was low (7,3 ± 5,3 g).

Because the recommended intakes of micronutrients are different for sex and age we evaluated the adequacy of nutrient intake using the following index: % adequacy = Eurpopean Reccomanded dietary allowances (RDA) for sex and age/estimated intake * 100. The results are shown in Table 2. With the exception of copper, there was a low intake of minerals, in particular calcium, iron and zinc. Also thiamin, riboflavin and niacin were also 25% below the European Recommended Dietary Allowances for sex and age [22].

The energy intake normalised for body weight (EI/BW -kcal/kg-) increased in patients with severe neurological impairment (r^2 ^= 0,37) while no correlations were found between EI/BW and feeding problems or impairment of self-feeding skills.

The EI/BW was significantly greater in patients with lower values of %BW and %IBW-H (r^2 ^= 0,37 and r^2 ^= 0,42 respectively).

The measured REE normalised for body weight (REE/BW -kcal/kg-) was higher in children with severe disabilities in comparison to other subjects.

Our study aimed to evaluate nutritional status, energy metabolism and food intake in children affected by drug resistant epilepsy. We hypothesised that RE can induce a nutritional status impairment.

In our study about 70 percent of patients had a moderate or severe neurological and self-feeding skills impairment.

Using some anthropometric indexes (%BW, %BH and %IBW-H) as criterion for the assessment of nutritional status, our results demonstrated the existence of nutritional status impairment. According to these criterions about 40% (applying %BW as index of nutritional status) resulted malnourished and 24% (applying %IBW-H) of children appeared wasted and only one patient resulted over nourished for both indexes. However, all the children had deficits in mean values of both measurements compared with reference values.

From these data it would seen that the identification of a nutritional impairment depends on which anthropometric criteria is used. In particular by just applying the %IBW-H index, children who are under 80% weight for age value would not be identified. This value is the safety nutritional cut-off that signifies the healthy nutritional status, therefore %BW should be used together with %IWH.

Of all of the variables we studied, the degree of neurological impairment was the best predictor of nutritional status, a finding that is consistent with those of a previous study focused on children with cerebral palsy [10].

The body composition assessment was used to better define the nutritional status in a subgroup of RE children. FM was reduced in undernourished patients and was well correlated with anthropometric indexes of nutritional status. There was a low fat free mass in all patients although it was more evident in patients with a severe neurological condition. This low FFM in all patients suggesting that even patients with normal anthropometric indexes could be undernourished. More than 50% of the children had a low BMD.

Macronutrients intake was unbalanced leading to a hyperproteic, hyperlipidic and hypoglucidic diet with a very low amount of fibre. Moreover the mean daily intake of calcium, iron, copper, tiamin, riboflavin and niacin was greatly reduced compared with European Recommended Dietary Allowances for sex and age [22].

On average, daily energy intake was lower than recommended values; however we found an unexpected lack of agreement between EI/BW and nutritional status because the EI/BW was higher in the more malnourished patients. An explanation could be that there was a higher REE/BW in the more malnourished children; REE/BW index was linked to the degree of neurological impairment.

Among the children in our study, those with the worst neurological condition were hypercatabolic. Although their EI/BW was higher, they needed more energy than they got from their energy intake, so leading to a malnutrition status.

Undernutrition has been recognized in other neurological diseases both in adults and in children [28-30]. The importance of malnutrition in patients with RE is related to the possible influence of nutritional status in the long-term prognosis, as well as to the predisposition to infections [31].

In conclusion our study showed that undernutrition is a frequent condition in RE children and it can be easy detected by anthropometric evaluation.

Further studies should be made in order to understand which of the anthropometric indexes better defines the nutritional status of these RE children. Furthermore they should undergo a routine evaluation of the body composition, so as to assess their nutritional status. If the total amount and regional distribution of fat lean body mass and bone mineral content is knows then a personalised diet and physical therapy program could be planned.

The use of standard predictive formulae for the evaluation of resting energy expenditure, has limited applications in this clinical group. However, by combining regular REE measurements with the analysis of the dietary intake, the specific energy requirements could be optimized and the macro, micro nutrient daily intake could be corrected.

Clearly there is much to be learnt to define optimal dietary advice for RE patients, and to define the relation between dietary components and indices of nutritional status.

## Abbreviations

%BH = percentage of ideal body height for sex and age at 50th percentile

%BW = percentage of ideal body weight for sex and age at 50th percentile

%IBW-H = percentage of ideal weight for height, sex, and age

BH = Body Height (cm)

BMC = bone mineral content (g)

BMI = Body Mass Index (kg/cm^2^)

BW = Body Weight (Kg)

DXA = Dual-energy X-ray absorptiometry

EI/BW = energy intake normalised for body weight (kcal/kg)

EI = Total energy intake (kcal/die)

FFM = fat free mass (Kg)

FM = fat mass (Kg)

RE = Refractory epilepsy

REE/BW = Resting energy expenditure normalised for body weight (kcal/kg)

REE = Resting energy expenditure (kcal/die)

**Table 2:** daily micronutrients intake and evaluation of adequacy intake in children with RE (n = 17)

Micronutrients	Mean (SD)	% adequacy (SD)
Calcium (mg/die)	519,7 (284,9)	51,0 (27,3)
Phosphate (mg/die)	708,2 (186,6)	69,9 (16,7)
Potassium (mg/die)	1594,1 (564,9)	83,6 (35,7)
Iron (mg/die)	6,2 (2,6)	63,7 (1,7)
Zinc (mg/die)	4,1 (1,9)	56,8 (1,5)
Copper (mg/die)	0,8 (0,4)	116,8 (62,2)
Thiamin (mg/die)	0,6 (0,2)	63,6 (22,1)
Riboflavin (mg/die)	0,9 (0,2)	74,1 (38,7)
Niacin (mg/die)	9,4 (6,2)	72,2 (46,4)
Vitamin A (mcg/die)	499,7 (279,7)	97,2 (52,1)
Vitamin C (mg/die)	53,8 (34,9)	116,4 (77,5)

% adequacy = recommend intake for sex and age/estimated intake * 100

## References

[B1] Mayer SA, Claassen J, Lokin J, Mendelsohn F, Dennis LJ, Fitzsimmons BF (2002). Refractory status epilepticus: frequency, risk factors, and impact on outcome. Arch Neurol.

[B2] Shields WD (2000). Catastrophic epilepsy in childhood. Epilepsia.

[B3] Rodriguez-Barrionuevo AC, Bauzano-Poley E, Rodriguez-Vives MA (1998). Diagnostic focus on the child with epilepsy and neuropsychological deterioration. Rev Neurol.

[B4] Artigas J (1999). Psychological manifestations of epilepsy in childhood. Rev Neurol.

[B5] Trier E, Thomas AG (1998). Feeding the disabled child. Nutrition.

[B6] Thommessen M, Riis G, Kase BF, LaREn S, Heiberg A (1991). Energy and nutrient intakes of disabled children: do feeding problems make a difference?. J Am Diet Assoc.

[B7] Richard D, Ferland J, Lalonde J, Samson P, Deshaies Y (2000). Influence of topiramate in the regulation of energy balance. Nutrition.

[B8] Lampl Y, Eshel Y, Rapaport A, Sarova-Pinhas I (1991). Weight gain, increased appetite, and excessive food intake induced by carbamazepine. Clin Neuropharmacol.

[B9] Baer MT, Kozlowski BW, Blyler EM, Trahms CM, Taylor ML, Hogan MP (1997). Vitamin D, calcium, and bone status in children with developmental delay in relation to anticonvulsant use and ambulatory status. Am J Clin Nutr.

[B10] Dahl M, Thommessen M, Rasmussen M, Selberg T (1996). Feeding and nutritional characteristics in children with moderate or severe cerebral palsy. Acta Paediatr.

[B11] Thommessen M, Kase BF, Riis G, Heiberg A (1991). The impact of feeding problems on growth and energy intake in children with cerebral palsy. Eur J Clin Nutr.

[B12] Lohman TG, Roche AF, Martorel R (1988). edn. Human Kinetics Books: Champagne Illinois. Anthropometric standardization Reference Manual.

[B13] Tanner JM, Whitehouse RH, Takaishi M (1966). Standards from birth to maturity for height, weight, height velocity, and weight velocity: British children, 1965. II. Arch Dis Child.

[B14] Waterlow JC (1973). Note on the assessment and classification of protein-energy malnutrition in children. Lancet.

[B15] Cole TJ, Donnet ML, Stanfield JP (1981). Weight-for-height indices to assess nutritional status – a new index on a slide-rule. Am J Clin Nutr.

[B16] Ramsey BW, Farrell PM, Pencharz P (1992). Nutritional assessment and management in cystic fibrosis: a consensus report. The Consensus Committee. Am J Clin Nutr.

[B17] Charlton CP, Buchanan E, Holden CE, Preece MA, Green A, Booth IW, Tarlow MJ (1992). Intensive enteral feeding in advanced cirrhosis: reversal of malnutrition without precipitation of hepatic encephalopathy. Arch Dis Child.

[B18] Uderzo C, Rovelli A, Bonomi M, Barzaghi A, Strada S, Balduzzi A, Pirovano L, Masera G (1996). Nutritional status in untreated children with acute leukemia as compared with children without malignancy. J Pediatr Gastroenterol Nutr.

[B19] Rolland-Cachera MF, Sempe M, Guilloud-Bataille M, Patois E, Pequignot-Guggenbuhl F, Fautrad V (1982). Adiposity indices in children. Am J Clin Nutr.

[B20] Isbell TR, Klesges RC, Meyers AW, Klesges LM (1991). Measurement reliability and reactivity using repeated measurements of resting energy expenditure with a face mask, mouthpiece, and ventilated canopy. J Parenter Enteral Nutr.

[B21] Weir JBV (1949). New methods for calculating metabolic rate with special to protein metabolism. Journal of Physiology.

[B22] Reports of the Scientific Committee for Food (1993). Nutrient and energy intakes for the European Community.

[B23] Frisancho AR (1981). New norms of upper limb fat and muscle areas for assessment of nutritional status. Am J Clin Nutr.

[B24] Tanner JM, Whitehouse RH (1962). Standards for subcutaneous fat in British children. Percentiles for thickness of skinfolds over triceps and below scapula. Br Med J.

[B25] Ellis KJ (1997). Body composition of a young, multiethnic, male population. Am J Clin Nutr.

[B26] Ellis KJ, Abrams SA, Wong WW (1997). Body composition of a young, multiethnic female population. Am J Clin Nutr.

[B27] Energy and protein requirements. WHO Technical Report Series 724.

[B28] Chong SK (2001). Gastrointestinal problems in the handicapped child. Curr Opin Pediatr.

[B29] Sullivan PB, Lambert B, Rose M, Ford-Adams M, Johnson A, Griffiths P (2000). Prevalence and severity of feeding and nutritional problems in children with neurological impairment: Oxford Feeding Study. Dev Med Child Neurol.

[B30] Hals J, Ek J, Svalastog AG, Nilsen H (1996). Studies on nutrition in severely neurologically disabled children in an institution. Acta Paediatr.

[B31] Chandra S, Chandra RK (1986). Nutrition, immune response and outcome. Prog Food Nutr Sci.

